# Pathogenesis of Pregnancy-Related Complications 1.0 and 2.0

**DOI:** 10.3390/ijms23063020

**Published:** 2022-03-11

**Authors:** Ilona Hromadnikova

**Affiliations:** Department of Molecular Biology and Cell Pathology, Third Faculty of Medicine, Charles University, 10000 Prague, Czech Republic; ilona.hromadnikova@lf3.cuni.cz; Tel.: +420-296511336

These Special Issue *IJMS* were dedicated to the major complications responsible for maternal and perinatal morbidity and mortality, such as gestational hypertension (GH), preeclampsia (PE), fetal growth restriction (FGR), gestational diabetes mellitus (GDM), preterm birth, and chronic venous disease.

These Special Issues provided an overview of the latest research on the molecular and cellular mechanisms associated with pregnancy-related complications, novel diagnostics/prognostics modalities, as well as on the long-term consequences of pregnancy-related complications, both for mothers and their offspring. In addition, to reflect the current state-of-the-art and actual needs in the field, several review studies dedicated to the placental toxicology, the cervical mucus barrier, and periodontitis were also implemented.

In our first Special Issue (closed on 29 February 2020), three reviews and five original papers were published altogether.

The review of Gerasimova et al. [1] summarized the role of protein misfolding and aggregation in the pathogenesis of PE. The toxic deposition of proteins, inclusive of amyloid-like aggregates, appear both in the placenta and body fluids of patients affected with PE and may serve as a potential biomarker of PE.

The review of Ziganshina et al. [2] conducted a survey on the changes of endothelial glycocalyx that result in endothelial dysfunction, playing a central role in clinical symptomatology of PE such as progressive endotheliosis, hypertension, tissue edema, disseminated intravascular coagulation syndrome, and impaired permeability of glomerular and hematoencephalic barriers.

The review of Alejandro et al. [3] was dedicated to GDM. Several important aspects involving screening, diagnosis, peripartum complications, and preclinical models of the disease, together with long-term maternal and neonatal outcomes, and the potential health risk associated with GDM persisting into the next generation were disscussed in detail.

Our research group [4] demonstrated that endothelial dysfunction appeared in a proportion of overweight women and in a proportion of women with central obesity. In addition, an association between the occurrence of endothelial dysfunction and altered expression profiles of miR-1-3p, miR-23a-3p, and miR-499a-5p in the whole peripheral blood of young and middle-aged women was detected.

Szilagyi et al. [5] introduced gene modules and clusters involved in the dynamic differentiation of villous cytotrophoblasts into the syncytiotrophoblasts and its impairment in preterm PE, which may be used for the early, non-invasive identification of women at risk of preterm PE and other placenta-mediated pregnancy complications.

Starodubtseva et al. [6] introduced seven urine *SERPINA1* (α1-antitrypsin) peptides as one of the most promising peptide biomarkers of PE. Urine *SERPINA1* peptides were associated with the destruction and degradation of thesyncytiotrophoblast membrane and, consequently, with the severity of the disease.

Our research group [7] described the altered postpartal expression profile of microRNAs associated with diabetes mellitus and cardiovascular and cerobrovascular diseases in the whole peripheral blood of mothers with a history of GDM. The screening of particular microRNAs may stratify a high-risk group of mothers with a history of GDM who might benefit from the implementation of early primary prevention strategies with the aim to decrease cardiovascular risk.

Ortega et al. [8] observed increased angiogenesis and lymphangiogenesis in the placental villi of women with chronic venous disease during gestation. Gene expression of CD31, podoplanin (D2-40), Flt-1, and PIGF in the placental villi and the number of vessels positive for CD31 and D2-40 were significantly increased in women with chronic venous disease.

In our second Special Issue (closed on 28 February 2021) nine reviews and six original papers were published altogether.

The review of Lacroix et al. [9] brought current knowledge on the cervicovaginal mucus barrier and its interaction with the microbiome in the physiological state and bacterial vaginosis. Special attention was paid to abnormalities of the cervical mucus plug and bacterial vaginosis associated with a higher risk of preterm birth.

The review of Szczuko et al. [10] discussed in detail the role of proinflammatory mediators of arachidonic (AA) and linoleic acid (LA) derivates in reproduction and pregnancy. The review described, among other topics, the role of AA and LA derivates in fetal growth and pregnancy-related complications such as GDM and PE.

The review of Todros et al. [11] summarized the role of proinflammatory cytokine macrophage migration inhibitory factor (MIF), a critical regulator of the innate immune response and a key factor regulating placental development in the pathogenesis of early and late PE.

The review of Matsubara et al. [12] was dedicated to the description of the role of exosomes in pathogenesis of PE. The review demonstrated the potential prediction of PE via the screening of microRNA profiles in blood exosomes and the regenerative abilities of exosomes derived from mesenchymal stem cells through the suppression of extravillous trophoblast apoptosis and the promotion of their invassiveness.

The clinical review of Ornoy et al. [13] introduced in-depth multiple risks to the embryo, fetus, course of gestation, and offspring associated with pregestational diabetes mellitus (PGDM) and gestational diabetes mellitus (GDM).

The review of Pankiewicz et al. [14] summarized recent advances in the pathogenesis of PE, with particular emphasis on defective uterine artery remodeling and the role of microRNAs. The update to the two-stage model of PE via the implementation of novel pathways leading to clinical manifestations in the second part of pregnancy was discussed.

The review of Ruszala et al. [15] discussed novel serum biomarkers involving galectins, growth differentiation factor-15, chemerin, omentin-1, osteocalcin, resistin, visfatin, vaspin, irisin, apelin, fatty acid-binding protein 4, fibroblast growth factor 21, and lipocalin-2 to predict the occurrence of GDM.

The aim of the review of Bendek et al. [16] was to come closer to the mechanisms involved in the association between periodontitis and GDM, which both contribute to systemic inflammation. Communication between periodontal and placental tissues via extracellular vesicles carrying non-coding RNA was discussed.

The review of Deval et al. [17] was dedicated to the description of the impact of toxic cerium dioxide nanoparticles used in various fields for industrial and commercial applications, on the placental barrier and pregnancy outcome.

Kelemu et al. [18] described the association between the single nucleotide polymorphism (SNP) rs311103, located in a GATA-binding site in a regulatory region on the X/Y chromosomes, and PE in pregnancies carrying male fetuses in Ethiopian women.

Pasternak et al. [19] reported the function and composition of high-density lipoproteins (HDL) among women with class A2 GDM and their fetuses. They observed the changes in HDL both in maternal and placental samples but not in cord samples.

Burzynska-Pedziwiatr et al. [20] performed the targeted metabolomic profiling in patients with GDM during gestation and postpartum period and revealed that the downregulation of arginine played a critical role in the development of GDM and in addition may also predict increased risk of type 2 diabetes mellitus.

Keckstein et al. [21] demonstrated altered expression of proinflammatory cytokines IL-7, IL-8, and IL-15 in placental tissues derived from patients with GDM, playing a role in the maintenance of a state of chronic low-grade inflammation. In addition, fetal sex-specific differences were detectable in all three cytokines.

Our research group [22] described the association between the expression of cardiovascular-disease-associated microRNAs in the whole peripheral blood and the incidence of prehypertension/hypertension, overweight/obesity, valve problems, and heart defects in children at the age of 3–11 years that were born from normal pregnancies and pregnancies complicated with GH, PE, FGR, GDM, and/or preterm birth.

Finally, Kedziora et al. [23] investigated the effect of PE on postpartum renal damage in a transgenic rat model. Almost all pathologies (albuminuria, glomerular, and tubular function) observed during PE recovered postpartum.

## Data Availability

Not applicable.
